# Developing and Piloting Suicide Prevention Training in Pediatric Primary Care

**DOI:** 10.1016/j.jaacop.2024.12.001

**Published:** 2024-12-11

**Authors:** John T. Parkhurst, Mallory Hilliard, Dana E. Hubbell, Andrea E. Spencer, Jennifer A. Hoffmann, Elizabeth Martinez-Charleston, Jeffrey L. Loughead, Aron Janssen

**Affiliations:** a Ann & Robert H. Lurie Children’s Hospital of Chicago, Chicago, Illinois; b Northwestern University Feinberg School of Medicine, Chicago, Illinois; c DePaul University, Chicago, Illinois

**Keywords:** suicide, medical education, training, primary care, community engagement

## Abstract

**Objective:**

Suicide prevention is an expected competency of pediatric primary care clinicians (PPCCs). Suicide prevention training should be tailored to the recipient’s setting, localized needs, and available models of care. This article describes a first step in community-participatory development and pilot evaluation of a suicide prevention training series for PPCCs.

**Method:**

Using guidance from community advisors and a survey of PPCCs (N = 81), we developed a pragmatic 3-part suicide prevention training series, consisting of a brief didactic (20 minutes), video demonstration (20 minutes), and simulated role play (30 minutes). The suicide prevention training series was piloted with 20 PPCCs to assess feasibility, acceptability, and PPCC confidence.

**Results:**

PPCCs who desired training in suicide risk identification (54%) and managing patients after risks were identified (67%), which were integrated into the training series. The training had high acceptability (mean ≥ 4.03 on the Acceptability of Intervention Measure) and feasibility (mean ≥ 3.73 on the Feasibility of Intervention Measure) across the 3 components. Participant confidence in screening, assessing, and safety planning increased 22.7% from baseline to post assessment (n = 18, *p* = .010, *d* = 1.23) and 23.2% at the 2-month follow up (n = 16, *p* = .010, *d* = 1.45).

**Conclusion:**

Suicide prevention training that is responsive to the challenges inherent to the pediatric primary care setting is feasible, acceptable, and increases confidence of PPCCs in conducting evidence-based suicide prevention interventions. Considerations for the practicality and opportunities for advancement in evaluation of suicide prevention training are explored.

**Diversity & Inclusion Statement:**

We worked to ensure that the study questionnaires were prepared in an inclusive way. One or more of the authors of this paper self-identifies as a member of one or more historically underrepresented sexual and/or gender groups in science. One or more of the authors of this paper self-identifies as a member of one or more historically underrepresented racial and/or ethnic groups in science. We actively worked to promote sex and gender balance in our author group. We actively worked to promote inclusion of historically underrepresented racial and/or ethnic groups in science in our author group. While citing references scientifically relevant for this work, we also actively worked to promote sex and gender balance in our reference list. While citing references scientifically relevant for this work, we also actively worked to promote inclusion of historically underrepresented racial and/or ethnic groups in science in our reference list. The author list of this paper includes contributors from the location and/or community where the research was conducted who participated in the data collection, design, analysis, and/or interpretation of the work.

## Plain language summary

This study developed and piloted a suicide prevention training for pediatric primary care clinicians using clinician input. The three-part training series included education, video demonstration, and role play for screening, assessing, and safety planning with youth at risk for suicide. The training was well received, and significantly increased clinician confidence in identifying and managing suicide risk. These findings suggest that practical suicide prevention training for clinicians can enhance their skills and confidence in delivering suicide prevention interventions.

The prevention of youth suicide requires a multi-pronged public health approach, inclusive of strategies delivered in primary care.^1^, ^2^, ^3^ Pediatric primary care clinicians (PPCCs) have a foundational role in the detection of mental health concerns and suicide risk,^4^ and are the most likely health care professionals to interact with a patient at risk for suicide.^5^^,^^6^ PPCCs,^7^^,^^8^ and their patients,^9^ largely accept that suicide prevention should be part of a pediatric primary care visit. Increasingly, there are recommendations to screen for suicidal ideation and behavior, and to formally assess to confirm risk, evaluate imminent danger, and create a plan, including the American Academy of Pediatrics 2022 Blueprint for Youth Suicide Prevention.^10^

Despite recognizing the importance of primary care suicide prevention strategies, 39% of PPCCs do not consistently screen for suicide risk, and only 27% of those who do screen for suicide use a suicide-specific measure.^5^ The most-often cited barrier to PPCCs engaging in suicide prevention is perceived lack of time.^5^^,^^11^, ^12^, ^13^ However, multiple examples demonstrate the feasibility of implementing effective suicide prevention practices in pediatric primary care settings.^13^, ^14^, ^15^, ^16^, ^17^ The contrast between PPCC perceptions and actual implementation of suicide prevention activities suggests a knowledge and confidence gap.^18^^,^^19^ Training PPCCs in suicide prevention strategies, including screening, assessment, and safety planning, can improve PPCC attitudes and confidence, and may ultimately prevent youth suicide.^3^^,^^4^^,^^18^^,^^20^

Pediatric primary care clinicians face unique challenges to implementation of suicide prevention strategies. Many (47.2%) PPCCs still work in independently owned, small- to medium-sized practices that may lack technological and health system resources (ie shared electronic health record, access to behavioral health services, or collaborative treatment models).^21^ Compared to adult primary care, primary presenting problems in pediatrics are often new (47.4% in pediatrics vs 27.9% in adults), placing a greater burden on PPCCs’ ability to assess and provide anticipatory guidance for many conditions.^22^ Finally, PPCCs must balance both patient and caregiver responses to suicide prevention strategies, including navigating confidentiality with adolescents and caregivers.

Existing suicide prevention trainings have not been developed for the context and challenges of the pediatric primary care setting,^23^ which requires a community-partnered approach that is responsive to the specific needs of community PPCCs and undermines historical health inequity.^24^ Given that training has not been designed to explicitly address specific barriers faced by PPCCs, participatory research is needed to tailor suicide prevention training to pediatric primary care.

The objective of this work was to advance the development of a suicide prevention training intervention for PPCCs in community practices. We engaged in the following 3 steps: (1) a targeted needs assessment, informed by engaging community advisors and surveying PPCCs to identify challenges, preferences, and context-dependent needs; (2) development of a training intervention informed by the needs assessment; and (3) pilot testing the training intervention to assess feasibility, acceptability, and effectiveness as measured by changes in PPCC confidence following the training intervention.

## Method

### Study Design

**Figure 1:**
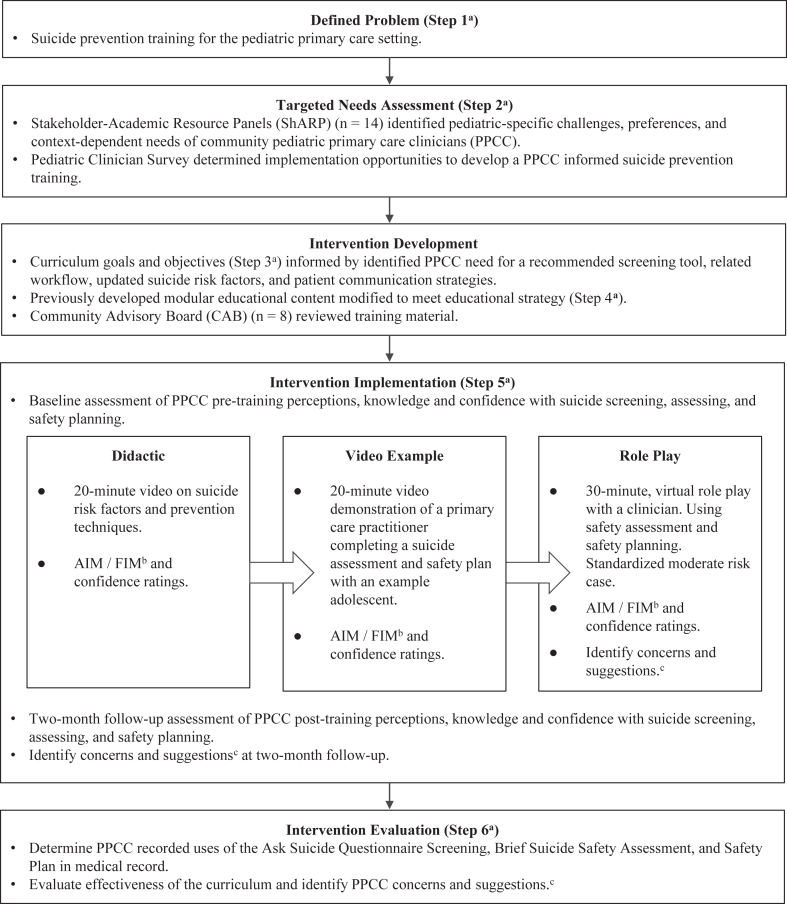
Development, Implementation, and Evaluation of Suicide Prevention Training Pilot ***Note:***^*a*^*Six steps identified from Kern’s approach to medical curriculum creation.* ^*b*^*Acceptability of Intervention Measure (AIM) / Feasibility of Intervention Measure (FIM).* ^*c*^*Assess pediatric primary care clinicians (PPCC) concerns about implementing suicide prevention practices, feedback about the training intervention, and suggestions for future suicide prevention initiatives.*

The development of a suicide prevention training intervention for PPCCs followed the Kern *et al.* 6-step approach to medical education curriculum creation,^25^ in which we did the following: (1) defined the problem; (2) engaged in targeted needs assessment; (3) created goals and objectives of education; (4) identified an educational strategy; (5) implemented education; and (6) evaluated the effectiveness of the education program. Figure 1 depicts the development. The problem was defined as inadequate suicide prevention training materials tailored to the pediatric primary care setting. The targeted needs assessment included both community advisory panels and surveys of PPCCs. The targeted needs assessment informed goals and objectives, core components, and an educational training strategy recommended by PPCCs. We implemented a prototype suicide prevention training series with PPCCs in our region, evaluating the acceptability, feasibility, and effectiveness of the training in improving provider confidence. The Institutional Review Board reviewed and approved this research study.

### Community Advisors

Community advisors were included as participants^26^ to provide insight into the formation of the research question and identifying the training and educational needs of community members. Community advisors were recruited across a large diverse Midwestern city spanning urban and suburban care settings, and represented health care providers in multiple health systems and independent community practices. Community advisors were identified and recruited via e-mails to community pediatric practices, academic training clinics, and hospital-based services that interact with patients at increased risk for suicide (eg, child abuse pediatrics, emergency department).

Initially, our team held 2 Stakeholder-Academic Resource Panels (ShARP), with 14 total community advisors participating. The urban-based ShARP included 8 advisors (5 PPCCs, 1 emergency department pediatrician, 1 emergency department social worker, and 1 child psychiatrist). A suburban-based ShARP panel included 6 PPCCs. The 2 ShARPs were facilitated by an independent consultant, with semi-structured interview prompts provided by the researchers to learn about current practices and needs related to suicide prevention. Following these 2 panels, the research team identified 8 PPCCs who had participated in a ShARP and were willing to further support the research team as a Community Advisory Board (CAB) for the study. The CAB met twice, and actively reviewed training material created by the study team.

### Pediatric Clinician Survey

To expand on the themes identified by community advisors, a cross-sectional 26-item survey was developed to better understand PPCC practices, attitudes, and resource needs for suicide prevention initiatives (Supplement 1, available online). Our team collaborated with Zero Suicide developers to incorporate standard needs assessment language and content about suicide prevention resources.^1^ The CAB reviewed and provided comments on survey items. PPCCs were recruited to complete the survey via email through the large (N = 600) Division of Community-based Primary Care Pediatrics listserv.

### Pilot Intervention Development

The objective of the pilot intervention was to develop suicide prevention training materials tailored to the pediatric primary care setting. Materials were developed with the recognition that those receiving training had likely had prior and ongoing clinical interactions with youth at risk for suicide. Our team included psychologists, psychiatrists, social workers, and 8 CAB members (community PPCCs) who supported the development of training materials. Our team had experience creating modular educational content for PPCCs both in independent practice and in a large hospital system. The intention was to create educational content/training that could be completed on demand, that was not time intensive, and that included multiple learning opportunities. Prior to the development of materials, our team planned to address 3 primary suicide prevention strategies: (1) suicide risk screening, (2) assessment of patients with identified suicide risk, and (3) the use of a safety planning intervention.

### Pilot Intervention

#### Participants and Recruitment

A pilot sample of 20 PPCCs were recruited and consented to complete the suicide prevention training intervention, along with pre- and post-training assessments. Participants were recruited via e-mail sent to survey pediatric clinician participants and partnership with the medical leadership at 2 Federally Qualified Health Centers (FQHCs) to assist with recruitment. Participants completed informed consent procedures and were nominally reimbursed for their time.

### Procedures

The training intervention was a 3-part series consisting of an on-demand didactic (20 minutes), an on-demand video demonstration (20 minutes), and a live telehealth role play (30 minutes). The order of the training elements remained consistent.

Assessments were completed both before and after training using REDCap.^27^ A REDCap survey followed each training element, and PPCCs completed a post-assessment survey 2 months after role play completion.

Pediatric clinicians received the pre-assessment survey by e-mail. Subsequently, participants received instructions to complete the on-demand didactic session and follow-up assessment. After completion of part 1, participants received a link to part 2, the video demonstration and corresponding follow-up assessment. Participants were instructed to complete the 2 training elements and assessments within 1 week. Participants were permitted to save and return to their work at any point.

After completion of the didactic and video demonstration, participants received a Microsoft Bookings link and instructions to schedule a 30-minute role play during the following week at a time of their choosing. Role play times were available each week at varied times for participants to self-select. If a participant completed the first 2 training elements but had not scheduled the role play, they were provided up to 3 e-mail reminders.

### Measures

Measures assessed knowledge, confidence, perceived acceptability of training tools, perceived feasibility of training elements, and changes to suicide prevention behavior in practice.

Perceived feasibility and acceptability of the training was assessed using the Acceptability of Intervention Measure (AIM) and Feasibility of Intervention Measure (FIM),^28^ which are both 4-item measures rated using a 5-point Likert scale (5 indicating strongly agree). Psychometric studies of these measures indicate strong validity, reliability, and utility in implementation assessment.^28^^,^^29^

Participant confidence was assessed using 3 items: “I am confident when completing a suicide assessment with my patient,” “I am confident when identifying suicide risk and triaging patients appropriately,” and “I am confident when completing a safety plan with my patient and communicating this with a caregiver.” Our team developed these items from prior PPCC survey efforts.^30^ Each item was rated on a 5-point Likert scale (5 indicating strongly agree). These 3 items were analyzed individually and summed to generate a total confidence score (range, 3-15).

At baseline, knowledge was assessed with a 5-item didactic and a 3-part low-, medium-, and high-risk vignette series (9 items) created for the study. The 5-item didactic and 3 items from the medium-risk vignette example were combined for a total pre-assessment knowledge score, as only the medium-risk case example was expected to increase because of training. At 2 months post-training, these 8 items (5-item didactic and 3-item medium-risk vignette) were combined for a total post-assessment knowledge score.

To assess participant behavior, we extracted use of the Ask Suicide Screening Questions (ASQ) among patients ≥10 years of age in the electronic health record for a subset of 8 pediatric clinicians who engaged in the training series. Pre-training data were collected from February to March 2022, and post-training data were collected from February to March 2023.

### Analysis

The pilot intervention data were analyzed using RStudio.^31^ Descriptive statistics were used to evaluate acceptability, feasibility, knowledge acquisition, and provider use of ASQ. The Friedman rank sum test was used to assess changes in participant confidence. The post hoc pairwise Wilcoxon test was performed with a Bonferroni-adjusted *p* value to determine statistically significant differences between participant confidence at pre-assessment, post-didactic, post-video, post-roleplay, and 2-month follow-up. The Cohen *d* was calculated with a 95% confidence interval to provide an estimate of effect size but not to determine significance. A Wilcoxon matched-pairs signed rank test was used to assess changes in participant use of the ASQ pre and post training.

## Results

### Community Advisory

The 14 ShARP participants identified high variability in screening practices (eg, measures used). Knowledge of risk and protective factors was identified as a strength for many; however, the procedures of completing a risk assessment were described as challenging to navigate. Barriers for risk assessment included lack of clarity about preferred tools and comfort with the use of these tools (eg, time for completion and integration into the electronic health record). Participants expressed a need for additional training and practice implementation for safety planning for patients at moderate risk for suicide, as patients at low and high risk were identified as being more straightforward to triage to care.

### Pediatric Clinician Survey

Of 600 community-based pediatric providers that were e-mailed, 121 participants agreed to take the survey and 81 participants completed the survey (13.5%, 81 of 600). Most participants were physicians that had trained in pediatrics (Table 1). Survey outcomes are reported in Table 2. PPCCs reported agreement (100%, 81 of 81) of their important role in suicide prevention. Of the 81 participants only 57% (46 of 81) identified any prior suicide prevention training. The majority of PPCCs believed that they were knowledgeable about suicide risk factors (77%, 62 of 81). Primary needs for training are reported in Table 3; these included communicating with patients about suicide (59%, 48 of 81), triage and management of risk (54%, 44 of 81), aftercare (62%, 50 of 81), and restriction of lethal means (73%, 59 of 81). The largest percentage of participants desired training in the form of brief video demonstration modules (51%, 41 of 81).

**Table 1:** Characteristics of Providers Who Responded to the Needs Assessment

Characteristic (N=81)	n (%)	Mean (SD, range)
Experience (years in practice)		19.1 (11.7, 1-39)
Graduate degree		
MD	76 (94)	
DO	3 (4)	
APN/DNP	1 (<1)	
Residency training		
Pediatrics	77 (95)	
Other	4 (5)	
Provider-estimated percentage of Medicaid patients seen		26.7 (37.1, 0-100)
Age at which assessing for suicide begins, y		
≥ 12	45 (56)	
10-12	30 (38)	
All ages	5 (6)	
Frequency of screening tool use		
Well child	50 (62)	
All visits	11 (13)	
Other	16 (20)	
Designated time	4 (5)

**Table 2:** Frequency of Needs Assessment Providers Who Stated Agreement With Suicide-Related Attitudes and Behaviors

Attitude characteristic (N = 81)	n (%)^a^
I believe suicide prevention is an important part of my professional role.	81 (100)
I am comfortable asking patients direct and open-ended questions about suicidal thoughts and behaviors.	71 (88)
I have the knowledge and training needed to recognize when a patient may be at elevated risk for suicide.	62 (77)
I am comfortable screening patients for suicide risk.	61 (75)
I have the knowledge and skills needed to screen patients for suicide risk.	59 (73)
I am familiar with the clinical workflows at my practice related to things such as safety planning.	47 (58)
I have received training related to suicide prevention.	46 (57)
I am current on the research of risk factors and patient features that place an individual at highest risk for suicide attempts or completion.	40 (49)
I have directly or indirectly interacted with a patient who ended their life by suicide.	26 (32)

**Behavior characteristic (N = 81)**	**n (%)**^a^

Use a suicide screening tool.	79 (98)
Use screening tools at well-child checks.	50 (62)
Consistently document a safety plan or safety contract with patients who identify suicidal ideation.	41 (51)
Their practice has created a safety plan or safety contract documentation.	32 (40)
Use screening tool at every visit.	11 (14)

Note: Response options were on a 5-point Likert scale ranging from strongly disagree to strongly agree. Providers responding agree and strongly agree were considered to be in agreement with the statement.

a n (%) Reflects the frequency and percentage of providers who stated agreement with the attitude or behavior.

**Table 3:** Frequency of Needs Assessment Providers Who Requested Each Type of Additional Education

Requested area of additional training/education (N = 81)	n (%)^a^
Reducing access to lethal means outside the care environment	59 (73)
Aftercare and follow-up	50 (62)
Communicating with patients about suicide	48 (59)
Identifying warning signs for suicide	44 (54)
Determining appropriate levels of care for patients at risk for suicide	44 (54)
Managing suicidal patients	40 (49)
Crisis response procedures and de-escalation techniques	39 (48)
Procedures for communicating about potentially suicidal patients	38 (47)
Suicide screening practices	36 (44)
Suicide risk assessment practices	36 (44)
Understanding and navigating ethical and legal considerations	35 (43)
Suicide prevention and awareness for patients	34 (42)
Family, caregiver, and community supports	34 (42)
Collaborative safety planning for suicide	33 (41)
Identifying risk factors for suicide	32 (40)
Suicide-specific treatment approaches	32 (40)
Epidemiology and the latest research findings related to suicide	29 (36)
Creating a safe physical environment for patients at risk for suicide	28 (35)
Policies and procedures within your work environment	21 (26)

Note: Providers were able to select as few or as many checkboxes as desired to designate which types of additional education they would like.

a n (%) Reflects frequency and percentage of providers who requested the additional education.

### Pilot Intervention Development

Based on the above results, we identified the following learning objectives that needed to be addressed by training: (1) identifying an optimal primary care screening tool and related workflows; (2) providing updated information about suicide risk profiles; and (3) addressing patient communication strategies. The 20-minute on-demand didactic session was developed to incorporate current suicide risk factors, a recommended suicide risk screening tool (Ask Suicide Screening Questions [ASQ]), and a recommended primary care screening workflow (https://ramp.luriechildrens.org/en/curriculum/).^32^

Given the reported need for training on communication strategies to be used with patients at risk for suicide, our expert team scripted a patient and pediatrician demonstration about how an ASQ, Brief Suicide Safety Assessment (BSSA), and Stanley Brown Safety Plan could be used in a pediatric primary care setting. This 20-minute video demonstration was recorded in a pediatric office, with a pediatrician and model patient actor (https://ramp.luriechildrens.org/en/watch-videos/).^33^ As per the ASQ Toolkit (www.nimh.nih.gov/ASQ),^34^ the BSSA is meant to be a guide for the clinician and therefore was not conducted verbatim in the video. For the sake of brevity, we chose not to include pediatrician communication with a caregiver.

A 30-minute virtual role play between the PPCC and a behavioral health specialist was the final training element after the didactic and video example. A session agenda, sample vignette, and copies of the BSSA and Stanley Brown Safety plan templates were e-mailed to the PPCC prior to the session (Supplement 2, available online). The expert team developed a clinical vignette of a 14-year-old adolescent with recent thoughts of death who felt that their family and friends would be better off without them and with thoughts of killing themselves in the past week, as screened with an ASQ. The behavioral health specialist read the vignette and asked the PPCC to engage in the BSSA and safety plan for this patient. The specialist provided simulated responses and direction for sections of the BSSA that may have been missed. The role play was used to respond to participant specific questions related to suicide prevention strategies.

### Pilot Intervention Results

The piloted training series was found to be feasible, acceptable, and confidence building by participants. Of 20 clinicians who initiated training, 18 completed the entire series. Table 4 represents demographic information about the participants. Thirteen of the PPCCs who completed the pilot intervention had participated in the prior needs assessment survey. PPCCs worked in diverse settings (independent primary care, academic training clinic, and FQHCs). These providers also estimated treating a sizable publicly insured group of patients (36.6%). There was minimal missing data across the total number of response fields for all participants (2.0%, 15 of 746). Missing data were replaced with the participants’ average response on the given measure. The median times between completion of each survey were consistent with procedures (pre-assessment–didactic: 0 days, didactic–video: 7 days, video–roleplay: 7 days, roleplay–follow-up: 1.9 months). On average, each participating PPCC required 2 e-mail prompts to complete all training elements as assigned.

**Table 4:** Characteristics of Providers Who Participated in the Training Series

Characteristic (N = 20)	n (%)	Mean (SD, range)
Experience (years in practice)		19.7 (11.5, 2-40)
Graduate degree		
MD	17 (85)	
DO	1 (5)	
PA	1 (5)	
APP	1 (5)	
Residency training		
Pediatrics	19 (95)	
Family medicine	1 (5)	
Provider-estimated percentage of Medicaid patients seen		36.6 (42.3, 0-95)
Practice type		
Primary care	11 (55)	
FQHC	6 (30)	
Academic clinic	3 (15)	

Note: APP = Advanced Practice Provider; FQHC = Federally Qualified Health Center.

All training cycle elements had high acceptability and feasibility on the AIM and FIM measures: didactic (AIM mean = 4.45, 95% CI = 4.18-4.72; FIM mean = 4.34, 95% CI = 4.02-4.66), video demonstration (AIM mean = 4.03, 95% CI = 3.71-4.35; FIM mean = 3.76, 95% CI = 3.40-4.12), and role play (AIM mean = 4.29, 95% CI = 3.98-4.60; FIM mean = 4.40, 95% CI = 4.11-4.69).

No significant changes were noted in confidence from pre-assessment to post-didactic or from pre-assessment to post-video demonstration (Table 5). However, participants reported improved confidence in suicide prevention strategies (*p* = .010, *d* = 1.23, 95% CI = 0.496-1.974) from pre-training (n = 20, mean = 10.10, SD = 2.34) to post–role play (n = 18, mean = 12.39, SD = 1.42). This increase in participant confidence was sustained at the 2-month follow-up from baseline (*p* = .010, *d* = 1.45, 95% CI = 0.642-2.265) (n = 16, mean = 12.44, SD = 1.09).

**Table 5:** Mean, Standard Deviation, Effect Size, and Confidence Interval for Provider-Rated Confidence, Acceptability, and Feasibility Following Each Training Element

Outcome	N	Pre-Assessment	N	Didactic	*d* [95% CI]	*p*	N	Video	*d* [95% CI]	*p*	N	Role play	*d* [95% CI]	*p*	N	2 Month	*d* [95% CI]	*p*
		Mean (SD)		Mean (SD)				Mean (SD)				Mean (SD)				Mean (SD)		
Confidence total^a^	20	10.10 (2.34)	20	11.20 (1.85)	0.52 [–0.129, 1.173]	.373	20	11.30 (1.87)	0.57 [–0.086, 1.220]	.147	18	12.39 (1.42)	1.23 [0.496, 1.974]	.010∗	16	12.44 (1.09)	1.45[ 0.642, 2.265]	.010∗
Suicide assessment	20	3.45 (0.89)	20	3.80 (0.62)	0.46 [–0.190, 1.107]	.650	20	3.80 (0.70)	0.44 [–0.209, 1.087]	.650	18	4.17 (0.38)	1.10 [0.379, 1.835]	.071	16	4.12 (0.50)	1.25 [0.459, 2.037]	.025∗
Suicide triage	20	3.45 (0.76)	20	3.85 (0.59)	0.59 [–0.064, 1.243]	.147	20	3.80 (0.70)	0.48 [–0.169, 1.130]	.263	18	4.11 (0.68)	0.91 [0.199, 1.622]	.035∗	16	4.25 (0.45)	1.34 [0.543, 2.141]	.025∗
Safety plan	20	3.20 (1.01)	20	3.55 (0.83)	0.38 [–0.265, 1.026]	1.000	20	3.70 (0.66)	0.59 [–0.065, 1.243]	.443	18	4.11 (0.58)	1.20 [0.465, 1.937]	.033∗	16	4.06 (0.44)	1.23 [0.441, 2.016]	.048∗
Acceptability			20	4.45 (0.58)			20	4.03 (0.68)	0.66 [0.006, 1.322]	.059	18	4.29 (0.63)	0.39 [–0.292, 1.076]	.501				
Feasibility			20	4.34 (0.68)			20	3.76 (0.77)	0.79 [0.128, 1.458]	.014∗	18	4.40 (0.59)	0.00 [0.677, 0.677]	1.000				

Note: Acceptability and feasibility were assessed using the Acceptability of Intervention and Feasibility of Intervention Measures, which are 4-item measures rated using a 5-point Likert scale (5 indicating strongly agree). Provider confidence was assessed using 3 items on a 5-point Likert scale (5 indicating strongly agree). Suicide assessment: “I am confident when completing a suicide assessment with my patient”; suicide triage: “I am confident when identifying suicide risk and triaging patients appropriately”; safety plan: “I am confident when completing a safety plan with my patient and communicating this with a caregiver.”

*p* Values were calculated to determine statistical significance using the Friedman rank sum test and the post hoc pairwise Wilcoxon test with a Bonferroni adjustment. Cohen d was calculated with a 95% CI to provide an estimate of the effect size.

∗*p* < .05; ∗∗*p* < .01; ∗∗∗*p* < .001.

a Confidence total reflects the 3 confidence items pooled.

Participant knowledge and response to the medium-risk case vignette did not change significantly from pre-assessment to post-assessment (pre-assessment knowledge mean = 3.75, 95% CI = 3.09-4.41; post-assessment knowledge mean = 3.69, 95% CI = 2.92-4.46).

Among 8 participants for whom an electronic record of ASQ data was accessible for unique patients (age 10+ years), only 4 providers had at least 1 administration of the measure. As a proportion of eligible cases, 3 providers increased ASQ use from the 2-month period pre-training to the 2-month period post-training. PPCC 1 (0 of 76 to 1 of 64), PPCC 2 (2 of 169 to 54 of 117), and PPCC 3 (1 of 236 to 4 of 215). One PPCC’s ASQ use minimally declined from pre-training to post-training (14 of 75 to 12 of 75). There was no significant difference in ASQ use for these 4 participants.

## Discussion

This study advances research into suicide prevention by using a community-engaged strategy to develop training for the pediatric primary care setting. Following Kern’s medical education curriculum development model,^25^ we created a 3-part training series that used a didactic, video example, and live role play. The curriculum was informed by a regional survey of pediatric primary care clinicians and community advisory panels.^26^ The piloted training series was found to be feasible, acceptable, and confidence building by a pilot sample of PPCCs working in diverse settings.

### Feasibility and Acceptability

The feasibility and acceptability of the curriculum was generally rated favorably. Participants reported that the video-demonstration training element was the least feasible. This finding contrasted with pediatric clinician pre-survey results that identified video-demonstration as a preferred mechanism for suicide prevention training. Although video-based suicide prevention training didactics have previously been used with reasonable uptake,^35^ our video example included “what to do” and “what not to do” demonstrations of a PPCC completing an ASQ, BSSA, and a safety plan with a young actor. Participants responded that the length of the video example made the completion of the ASQ and BSSA seem unrealistic in practice. The participant feedback about the length of the video is interesting, as we specifically approached the administration of the BSSA in a pragmatic way, not including each potential line of questioning that could be pursued.

After receiving feedback from our participants, we edited the video-demonstration, taking out the ASQ (creating a separate, optional ASQ video example) and the “what not to do” sections. This edit reduced the video from 20 minutes to 13 minutes in length. Shortening the video length or creating multiple brief videos may improve the acceptability and feasibility of the video example in the future. However, this may not affect PPCC perception of time to complete suicide prevention strategies, as even our pragmatic demonstrations may seem unrealistic for the time available to primary care clinicians. Because time to implement suicide prevention activities is a frequently cited barrier,^5^^,^^11^, ^12^, ^13^ more work is needed to determine practical administration times for suicide assessment and safety planning to increase PPCC engagement in suicide prevention efforts.

### Participant Confidence and Knowledge

Participant confidence increased incrementally across the training series, with significant differences (*p* = .010) achieved after the post-training and sustained at the 2-month follow-up. Interestingly, the standard deviation of the mean confidence scores decreased across the training series, suggesting a wider disparity in participant confidence ratings at the outset of the training, with high confidence and consistency among participants post-training. Several of our pilot study participants were highly confident in suicide prevention strategies prior to the training. This affected our understanding of growth in confidence between training elements, especially in a small sample.

Medical education literature has previously identified role plays and simulations as effective in building physician confidence.^20^^,^^36^^,^^37^ The role play element may have been the most potent training element in this current effort, given that PPCC confidence in use of suicide prevention strategies changed significantly following the role play. However, more work is needed to find practical ways to incorporate role plays or patient simulation opportunities in pediatric primary care. For example, training practice champions, video simulation with branching response logic, or virtual reality^38^ could be used to facilitate simulated patient experiences for primary care practices in the community. More broadly, suicide prevention training and engagement may be enhanced by incorporating behavioral nudging^39^ or creating a collaborative care consultation model to engender PPCC confidence in delivering suicide prevention strategies and to provide patients access to resources.^40^, ^41^, ^42^

The training series did not have a significant impact on participant knowledge, which was likely due to a high floor effect for the relatively few knowledge questions. More curiously, participants did not improve in their responses to the management of a medium-risk patient vignette. This suggests that our training may not have effectively addressed participant understanding of how to triage challenging presentations of suicide risk. Because of this finding, the didactic portion now incorporates an additional medium-risk vignette.

### Participant Behavior

This study was undertaken to improve the effectiveness of suicide prevention training delivered in pediatric primary care. Although assessing proxy mechanisms of training effectiveness (eg, provider confidence) is a useful and common route for evaluating medical education,^3^^,^^43^ researchers must also assess the impact of training on physician behavior. We attempted to measure change in ASQ screening for a subset of 8 participants in our study. In attempting to detect changes in ASQ use, we identified challenges related to inconsistent documentation strategies by participants for suicide screening in the medical record (eg, in flowsheet or in provider notes). After identifying this, our team consolidated a suicide screening toolkit in a centralized location within the medical record to assess behavioral PPCC behavior going forward more effectively.

### Future Directions

The “push and pull” between time demands in pediatric primary care and the desire to have a rigorous approach to suicide prevention in pediatric primary care remains the greatest challenge that training programs must address, balancing rigor and practicality. Future directions should include a more robust trial of the training intervention delivered to PPCCs with emphasis on detecting behavioral outcomes that may result in suicide prevention. Furthermore, the suicide prevention training elements should be individually assessed and optimized to determine the training content, medium, and length that have the greatest impact on physician confidence and behavior. The goal of training is the most powerful content, efficiently delivered, resulting in provider engagement in suicide prevention activities.

Finally, we identified the need to provide enhanced lethal means counseling, including safe firearm and medication storage, with suicide prevention training.^4^^,^^44^ Lethal means safety counseling was identified as an area of need within the PPCC survey, as there is an established link between suicide completion and access to lethal means, especially firearms.

Our study team was intentional about engagement with community partners to develop suicide prevention training. This community-engaged approach should enhance generalizability for PPCCs while, importantly, enhancing perspectives of diversity and health equity.^45^ Despite the effort to include diverse community perspectives and to ensure that PPCCs working with minoritized populations participated in the pilot training, our work is limited to a localized sample. Furthermore, the community survey response rate of 13.5% is a limitation, as well as our pilot intervention sample of 20 participants. Thus, we cannot be confident that the PPCCs who completed the training were fully reflective of all PPCC practice settings, or that the information will generalize geographically. The PPCCs who engaged in our pilot study worked in diverse settings but were marginally reimbursed and self-selected into participation, which may have an impact on future dissemination.

Although this study demonstrated the feasibility and acceptability of the intervention as well as increased PPCC confidence in suicide risk management, further research is needed to determine whether this brief pragmatic approach to suicide risk management can increase PPCCs’ rates of suicide screening and assessment or can enhance the accuracy of PPCCs’ risk classification. Although we attempted to assess participant behavior change through the EHR, we could not systematically locate ASQ records because of variable participant reporting practices. As such, the effectiveness of our training approach requires further testing.

Successful suicide prevention will require the engagement of the large pediatric primary care clinician workforce. Many of these PPCCs work in small practice settings that may not have access to training and that have contextual barriers to engagement in suicide prevention activities. Engaging PPCCs in community participatory research to develop training can result in acceptable, feasible, and confidence-enhancing attitudes toward delivering suicide prevention strategies.

## CRediT authorship contribution statement

**John T. Parkhurst:** Writing – review & editing, Writing – original draft, Supervision, Project administration, Methodology, Investigation, Formal analysis, Conceptualization. **Mallory Hilliard:** Writing – original draft, Project administration. **Dana E. Hubbell:** Writing – original draft, Resources, Project administration, Formal analysis, Data curation. **Andrea E. Spencer:** Writing – original draft. **Jennifer A. Hoffmann:** Writing – review & editing. **Elizabeth Martinez-Charleston:** Project administration, Data curation. **Jeffrey L. Loughead:** Writing – review & editing, Investigation, Conceptualization. **Aron Janssen:** Writing – review & editing.
