# Supplementary material for: Developing and Piloting Suicide Prevention Training in Pediatric Primary Care
Source: JAACAP Open. 2024 Dec 11;3(3):538–47. doi: 10.1016/j.jaacop.2024.12.001 (PMC12414303; doi:10.1016/j.jaacop.2024.12.001)
Supplement: Supplement 1 [file mmc1.pdf]

Saving Lives Through Screening

You are being asked to take part in this study because of your familiarity with common mental health concerns and suicide risk assessment that presents in primary care. If you choose to participate, you will complete a survey. This survey will help us learn more about current practice, training needs, and gaps in care for suicide risk assessment in primary care. It will take about 5 minutes to complete.

Participating in this study is voluntary. You do not have to take this survey if you do not want to. The answers that you provide in the survey may still be used if you stop the survey and do not finish. You can skip questions that you do not want to answer or stop the survey at any time.

The survey is anonymous, and no one will be able to link your answers back to you once the data is collected. Your name will initially be used to track your data but be replaced with a numerical code for any dissemination efforts.

For participation in this study you will be randomly entered to win one of two $50 Visa gift cards. The information gained from this survey will help the researchers train and adapt practices to most effectively assess for suicide risk in pediatric primary care.

You indicate your voluntary agreement to take part in this research study by completing this survey.

Please enter your name:

____
(Last, First)

Email:

____
(This will only be used for gift card distrution if you are randomly selected.)

Please enter your graduate degree:

◯ MD
◯ DO
◯ Advanced Nurse Practitioner (e.g., APN, DNP)
◯ Physician's Assistant
◯ Other

If other, describe:

____

Your training was in:

◯ Pediatrics
◯ Family Medicine
◯ Medicine/Pediatrics
◯ Other

Other:

____

What year did you complete your training?

____

Are you board certified?

◯ Yes ◯ No

Please enter the number of years you have been practicing:

____

Please enter the zip code of where you practice:

____

Please indicate the estimated percentage of public aid patients you treat in your practice:

____

Definitions

Risk factors: Characteristics that precede and are associated with a higher likelihood of suicidal thoughts and behaviors.

Suicide risk assessment: Estimating the likelihood for a person to attempt suicide when risks for suicide are present.

Suicidal thoughts and behaviors (STB): Includes suicidal ideation, suicide attempt, and suicide death.

Suicide: Death caused by injurious behavior to the self with an intent to die.

Suicide attempt: A non-fatal, injurious behavior to the self with an intent to die; might not result in injury.

Suicidal ideation: Thinking about, considering, or planning suicide.

Prevention

	Strongly Disagree	Disagree	Neutral	Agree	Strongly Agree
I believe suicide prevention is an important part of my professional role.	◯	◯	◯	◯	◯
I have received training related to suicide prevention.	◯	◯	◯	◯	◯

I have directly or indirectly interacted with a patient who ended their life by suicide.

◯ Yes, it has happened once.
◯ Yes, it has happened more than once.
◯ No.
◯ I don't know.

Risk factor identification

	Strongly Disagree	Disagree	Neutral	Agree	Strongly Agree
I have the knowledge and training needed to recognize when a patient may be at elevated risk for suicide.	◯	◯	◯	◯	◯
I am current on the research of risk factors and patient features that place an individual at highest risk for suicide attempts or completion.	◯	◯	◯	◯	◯
I am comfortable asking patients direct and open-ended questions about suicidal thoughts and behaviors.	◯	◯	◯	◯	◯

Suicide screening tools

	Strongly Disagree	Disagree	Neutral	Agree	Strongly Agree
I have the knowledge and skills needed to screen patients for suicide risk.	◯	◯	◯	◯	◯
I am comfortable screening patients for suicide risk.	◯	◯	◯	◯	◯

Which of the following tools, screening and assessment instruments, or rubrics, if any, do you use? (Select all that apply)

- ☐ Asking Suicide-Screening Questions (ASQ).
- ☐ Beck's Suicide Intent Scale (SIS).
- ☐ Columbia Suicide Severity Rating Scale (C-SSRS).
- ☐ National Suicide Lifeline Risk Assessment Standards.
- ☐ Patient Health Questionaire-2 (PHQ-2).
- ☐ Patient Health Questionaire-9 (PHQ-9).
- ☐ Risk Assessment Matrix (RAM).
- ☐ Risk of Suicide Questionnaire (RSQ).
- ☐ Risk Formulation with Risk Status and Risk State.
- ☐ SAFE-T.
- ☐ Suicide to Hope.
- ☐ Suicide Ideation Questionnaire (SIQ or SIQ-JR).
- ☐ A tool, instrument, or rubric developed by my organization.
- ☐ A different tool, instrument, or rubric.

Please describe the different tool, instrument, or rubic you use:

____

At what age do you begin assessing for suicide:

- ◯ All ages.
- ◯ ≥10.
- ◯ 10-12.
- ◯ ≥12.

How frequently do you use a screening tool to assess suicidality?

- ◯ At every visit.
- ◯ At well child checks.
- ◯ At designated points yearly (e.g.twice yearly).
- ◯ Other.

If other, please describe:

____

How are suicide screening measures completed?

◯ Verbally asked of the patient without parent.
◯ Verbally asked of the patient with parent.
◯ Completed in written form during visit.
◯ Completed in electronic form prior to the visit.
◯ Other.

Other, please describe:

____

Providing care to patients at risk

	Strongly Disagree	Disagree	Neutral	Agree	Strongly Agree
I am familiar with the clinical workflows at my practice related to things such as safety planning.	◯	◯	◯	◯	◯
My practice has created a safety plan or safety contact documentation.	◯	◯	◯	◯	◯
I consistently document a safety plan or safety contract with patients who identify suicidal ideation.	◯	◯	◯	◯	◯

In which of the following areas, if any, would you like more training, resources, or support? (select all that apply)

	Training/Education	Resources/Support
Suicide prevention and awareness for patients.	☐	☐
Epidemiology and the latest research findings related to suicide.	☐	☐
Identifying warning signs for suicide.	☐	☐
Communicating with patients about suicide.	☐	☐
Suicide screening practices.	☐	☐
Identifying risk factors for suicide.	☐	☐
Suicide risk assessment practices.	☐	☐
Determining appropriate levels of care for patients at risk for suicide.	☐	☐
Crisis response procedures and de-escalation techniques.	☐	☐
Managing suicidal patients.	☐	☐
Collaborative safety planning for suicide.	☐	☐
Suicide-specific treatment approaches.	☐	☐
Aftercare and follow-up.	☐	☐
Family, caregiver, and community supports.	☐	☐
Procedures for communicating about potentially suicidal patients.	☐	☐
Understanding and navigating ethical and legal considerations.	☐	☐
Policies and procedures within your work environment.	☐	☐
Staff roles and responsibilities within your work environment.	☐	☐
Reducing access to lethal means outside the care environment.	☐	☐
Creating a safe physical environment for patients at risk for suicide.	☐	☐

What would be most beneficial way to improve consistent use of suicide screening tools in community pediatrics. ((Check all that apply))

☐ Additional training opportunities on the benefits/evidence for screening.
☐ Video demonstration or live training on how to complete screening in office.
☐ Financial targets linked to suicide screening.
☐ Identifying practice champions to support/help triage concerns in the pediatric office.
☐ Other.

If other, please describe:

____

I would be interested in being trained as a practice champion to prevent suicide in our community?

◯ Yes
◯ No

Do you have any comments about your clinical experience with suicide risk assessment that you would like to share with the researchers?
