# Supplementary material for: Developing and Piloting Suicide Prevention Training in Pediatric Primary Care
Source: JAACAP Open. 2024 Dec 11;3(3):538–47. doi: 10.1016/j.jaacop.2024.12.001 (PMC12414303; doi:10.1016/j.jaacop.2024.12.001)
Supplement: Supplement 2 [file mmc2.pdf]

Resources Advancing Mental Health in Pediatrics

RAMP

John Parkhurst, PhD
Mallory Hilliard, LCSW, CADC

Saving Lives Through Screening - Role Play Agenda

1. Introductions and review of agenda (5 min)
2. Participate in a safety assessment role play based on a real scenario (10 min)
    a. Employ the BSSA to support risk assessment
3. Employ the safety plan to support intervention and disposition of patient (10 min)
4. Troubleshoot issues in your practice (5 min)
    a. Discuss EPIC or EHR integration

Disclaimer: This document is for educational or informational purposes only and is not intended to substitute in any way for medical treatment, advice, or diagnosis by a healthcare professional. Please contact your provider for specific healthcare advice. Visit Lurie Children's at https://www.luriechildrens.org/en/privacy-legal-information/ to read our full Terms and Conditions.

© 2021 Ann & Robert H. Lurie Children's Hospital of Chicago

Ann & Robert H. Lurie Children's Hospital of Chicago
Pritzker Department of Psychiatry and Behavioral Health

Kai is a 14 y/o youth with no prior psychiatric history or treatment who presents today with his mother for an evaluation of daily headaches that started several months ago. Kai is given the ASQ as part of standard patient care and proves the following answers on the ASQ:

-"In the past few weeks, have you wished you were dead?"-No

-"In the past few weeks, have you felt that you or your family would be better off if you were dead?"-Yes

-"In the past week, have you been having thoughts about killing yourself?-Yes

-"Have you ever tried to kill yourself?"-No

"Are you having thoughts of wanting to kill yourself now?"-No

In talking with Kai individually, he denies current active suicidal ideations or thoughts of wanting to harm himself. He states that last week he wanted to die "because my dad took my phone away because I got a D on a test and I was talking back to him," but denied any specific plan or intent to end his life. He notes that a few times per week he feels that people would be better off without him and that no one would miss him. Shares that he has had some issues with her peers, with a few kids bullying Kai at school due to finding out about his parents' divorce. He reports a history of "scratching" his arms when he is upset, but denies engaging in this behavior currently and there are no visible marks on his arms upon examination.

He states that he talked to his school counselor a few weeks ago "because my parents are getting a divorce and kids are making fun of me because of it," and said it was "sort of" helpful. Kai shares that he talked to the counselor because one of his friends thought it might be a good idea because Kai appeared sadder at school.

In meeting with Kai's mom, mom reports that for the past several months, coinciding when Kai's parents began divorce proceedings, Kai has been more irritable, seems sad for no reason, and does not want to engage in extracurriculars as much. Also began reporting headaches around this time. Mom notes that Dad has noted similar observations when Kai is with him. Denies any firearms at home and reports that medications are easily accessible at home.

Brief Suicide Safety **Assessment**

asQ Ask Suicide-Screening Questions

What to do when a pediatric patient screens positive for suicide risk:

- Use after a patient **(8 - 24 years)** screens positive for suicide risk on the asQ
- Assessment guide for mental health clinicians, MDs, NPs, or PAs
- Prompts help determine disposition

1 Praise patient *for discussing their thoughts*

"I'm here to follow up on your responses to the suicide risk screening questions. These are hard things to talk about. Thank you for telling us. I need to ask you a few more questions."

2 Assess the patient *(If possible, assess patient alone depending on developmental considerations and parent willingness.)*

Review patient's responses from the asQ

Frequency of suicidal thoughts

Determine if and how often the patient is having suicidal thoughts.

Ask the patient: "In the past few weeks, have you been thinking about killing yourself?" **If yes, ask:** "How often?" (once or twice a day, several times a day, a couple times a week, etc.) "When was the last time you had these thoughts?"

"Are you having thoughts of killing yourself right now?" **(If "yes," patient requires an urgent/ STAT mental health evaluation and cannot be left alone. A positive response indicates imminent risk.)**

Suicide plan

Assess if the patient has a suicide plan, regardless of how they responded to any other questions (ask about method and access to means).

Ask the patient: "Do you have a plan to kill yourself?" **If yes, ask:** "What is your plan?" **If no plan, ask:** "If you were going to kill yourself, how would you do it?"

Note: If the patient has a very detailed plan, this is more concerning than if they haven't thought it through in great detail. If the plan is feasible (e.g., if they are planning to use pills and have access to pills), this is a reason for greater concern and removing or securing dangerous items (medications, guns, ropes, etc.).

Past behavior

Evaluate past self-injury and history of suicide attempts (method, estimated date, intent).

Ask the patient: "Have you ever tried to hurt yourself?" "Have you ever tried to kill yourself?"

If yes, ask: "How? When? Why?" and assess intent: "Did you think [method] would kill you?" "Did you want to die?" (for youth, intent is as important as lethality of method) **Ask:** "Did you receive medical/psychiatric treatment?"

Note: Past suicidal behavior is the strongest risk factor for future attempts.

Symptoms — Ask the patient about:

Depression: "In the past few weeks, have you felt so sad or depressed that it makes it hard to do the things you would like to do?"

Anxiety: "In the past few weeks, have you felt so worried that it makes it hard to do the things you would like to do or that you feel constantly agitated/on-edge?"

Impulsivity/Recklessness: "Do you often act without thinking?"

Hopelessness: "In the past few weeks, have you felt hopeless, like things would never get better?"

Anhedonia: "In the past few weeks, have you felt like you couldn't enjoy the things that usually make you happy?"

Isolation: "Have you been keeping to yourself more than usual?"

Irritability: "In the past few weeks, have you been feeling more irritable or grouchier than usual?"

Substance and alcohol use: "In the past few weeks, have you used drugs or alcohol?" **If yes, ask:** "What? How much?"

Sleep pattern: "In the past few weeks, have you had trouble falling asleep or found yourself waking up in the middle of the night or earlier than usual in the morning?"

Appetite: "In the past few weeks, have you noticed changes in your appetite? Have you been less hungry or more hungry than usual?"

Other concerns: "Recently, have there been any concerning changes in how you are thinking or feeling?"

Social Support & Stressors

(For all questions below, if patient answers yes, ask them to describe.)

Support network: "Is there a trusted adult you can talk to? Who? Have you ever seen a therapist/counselor?" **If yes, ask:** "When?"

Family situation: "Are there any conflicts at home that are hard to handle?"

School functioning: "Do you ever feel so much pressure at school (academic or social) that you can't take it anymore?"

Bullying: "Are you being bullied or picked on?"

Suicide contagion: "Do you know anyone who has killed themselves or tried to kill themselves?"

Reasons for living: "What are some of the reasons you would NOT kill yourself?"

Brief Suicide Safety Assessment

3 Interview patient & parent/guardian together

If patient is ≥ 18 years, ask patient's permission for parent/guardian to join.

Say to the parent: "After speaking with your child, I have some concerns about his/her safety. We are glad your child spoke up as this can be a difficult topic to talk about. We would now like to get your perspective."

- "Your child said… (reference positive responses on the asQ). Is this something he/she shared with you?"
- "Does your child have a history of suicidal thoughts or behavior that you're aware of?" **If yes, say:** "Please explain."
- "Does your child seem:
  - o Sad or depressed?"
  - o Anxious?"
  - o Impulsive? Reckless?"
  - o Hopeless?"
  - o Irritable?"
  - o Unable to enjoy the things that usually bring him/her pleasure?"
  - o Withdrawn from friends or to be keeping to him/herself?"
- "Have you noticed changes in your child's:
  - o Sleeping pattern?"
  - o Appetite?"
- "Does your child use drugs or alcohol?"
- "Has anyone in your family/close friend network ever tried to kill themselves?"
- "How are potentially dangerous items stored in your home?" (e.g. guns, medications, poisons, etc.)
- "Does your child have a trusted adult they can talk to?" (Normalize that youth are often more comfortable talking to adults who are not their parents)
- "Are you comfortable keeping your child safe at home?"

At the end of the interview, ask the parent/guardian: "Is there anything you would like to tell me in private?"

4 Make a safety plan with the patient

Include the parent/guardian, if possible.

Create a safety plan for managing potential future suicidal thoughts. A safety plan is different than making a "safety contract"; asking the patient to contract for safety is NOT effective and may be dangerous or give a false sense of security.

Say to patient: "Our first priority is keeping you safe. Let's work together to develop a safety plan for when you are having thoughts of suicide."

Examples: "I will tell my mom/coach/teacher." "I will call the hotline." "I will call _________ ."

Discuss coping strategies to manage stress (such as journal writing, distraction, exercise, self-soothing techniques).

Discuss means restriction (securing or removing lethal means): "Research has shown that limiting access to dangerous objects saves lives. How will you secure or remove these potentially dangerous items (guns, medications, ropes, etc.)?"

Ask safety question: "Do you think you need help to keep yourself safe?" (A "no" response does not indicate that the patient is safe; but a "yes" is a reason to act immediately to ensure safety.)

5 Determine disposition

After completing the assessment, choose the appropriate disposition plan. *If possible, nurse should follow-up with a check-in phone call (within 48 hours) with all patients who screened positive.*

- ❑ **Emergency psychiatric evaluation:** **Patient is at imminent risk for suicide (current suicidal thoughts).** Send to emergency department for extensive mental health evaluation (unless contact with a patient's current mental health provider is possible and alternative safety plan for imminent risk is established).
- ❑ **Further evaluation of risk is necessary:** Review the safety plan and send home with a mental health referral as soon as patient can get an appointment (preferably within 72 hours).
- ❑ **Patient might benefit from non-urgent mental health follow-up:** Review the safety plan and send home with a mental health referral.
- ❑ **No further intervention is necessary at this time.**

For all positive screens, follow up with patient at next appointment.

6 Provide resources to all patients

- **24/7 National Suicide Prevention Lifeline 1-800-273-TALK (8255) En Español: 1-888-628-9454**
- **24/7 Crisis Text Line: Text "HOME" to 741-741**

STANLEY - BROWN SAFETY PLAN

STEP 1: WARNING SIGNS:

1. ______________________________
2. ______________________________
3. ______________________________

STEP 2: INTERNAL COPING STRATEGIES – THINGS I CAN DO TO TAKE MY MIND OFF MY PROBLEMS WITHOUT CONTACTING ANOTHER PERSON:

1. ______________________________
2. ______________________________
3. ______________________________

STEP 3: PEOPLE AND SOCIAL SETTINGS THAT PROVIDE DISTRACTION:

1. **Name:** ______________________ **Contact:** ______________________

2. **Name:** ______________________ **Contact:** ______________________

3. **Place:** ______________________ **4. Place:** ______________________

STEP 4: PEOPLE WHOM I CAN ASK FOR HELP DURING A CRISIS:

1. **Name:** ______________________ **Contact:** ______________________

2. **Name:** ______________________ **Contact:** ______________________

3. **Name:** ______________________ **Contact:** ______________________

STEP 5: PROFESSIONALS OR AGENCIES I CAN CONTACT DURING A CRISIS:

1. **Clinician/Agency Name:** ______________________ **Phone:** ______________________

Emergency Contact : ______________________

2. **Clinician/Agency Name:** ______________________ **Phone:** ______________________

Emergency Contact : ______________________

3. **Local Emergency Department:** ______________________

Emergency Department Address: ______________________

Emergency Department Phone : ______________________

4. **Suicide Prevention Lifeline Phone:** 1-800-273-TALK (8255)

STEP 6: MAKING THE ENVIRONMENT SAFER (PLAN FOR LETHAL MEANS SAFETY):

1. ______________________________
2. ______________________________

The Stanley-Brown Safety Plan is copyrighted by Barbara Stanley, PhD & Gregory K. Brown, PhD (2008, 2021).
Individual use of the Stanley-Brown Safety Plan form is permitted. Written permission from the authors is required for any changes to this form or use of this form in the electronic medical record. Additional resources are available from www.suicidesafetyplan.com.

Stanley-Brown
Safety Planning Intervention
